# Multifocal intraocular lens exchange to monofocal for the management of neuroadaptation failure

**DOI:** 10.1186/s40662-022-00311-4

**Published:** 2022-11-01

**Authors:** Olena Al-Shymali, Jorge L. Alió del Barrio, Colm McAlinden, Mario Canto, Laura Primavera, Jorge L. Alio

**Affiliations:** 1 Cornea, Cataract and Refractive Surgery Unit, Vissum (Miranza Group), Alicante, Spain; 2 Division of Ophthalmology, School of Medicine, Universidad Miguel Hernández, Alicante, Spain; 3 Department of Ophthalmology, Royal Gwent Hospital, Newport, UK; 4 Vissum Miranza Alicante, Universidad Miguel Hernandez, Calle Cabañal 1, 03016 Alicante, Spain

**Keywords:** Multifocal intraocular lenses, Multifocal intraocular lens explantation, Monofocal intraocular lens, Dissatisfaction after multifocal intraocular lens implantation, Neuroadaptation failure, Patient satisfaction

## Abstract

**Background:**

The aim of this study was to evaluate visual, refractive, quality of vision, visual function and satisfaction of multifocal intraocular lens (MF-IOL) exchange with a monofocal IOL (MNF-IOL) in dissatisfied patients following MF-IOL implantation.

**Methods:**

This was a retrospective case series. Bilateral IOL exchange (MF-IOL to MNF-IOL) was performed in 13 patients (26 eyes) with neuroadaptation failure. Questionnaires including the Quality of Vision (QoV), Visual Function Index (VF-14 and Rasch-revised VF-8R version), and a satisfaction questionnaire were used.

**Results:**

The mean time for IOL exchange was 15 months. The corrected distance visual acuity (CDVA) improved from 20/26 to 20/23 (*P* = 0.028). The uncorrected near visual acuity (UNVA) worsened after exchange from 20/47 to 20/62 (*P* = 0.024). QoV scores improved significantly across all three subscales after exchange. Visual function for far distance improved with a change in VF-14 score from 74.2 ± 24.8 to 90.9 ± 9.1 (*P* = 0.03). The VF-8R score showed worsening although not statistically significant. Near vision spectacle independence was totally or partially lost in all cases. Ten patients (77%) reported they would not repeat the lens exchange. Safety and efficacy indices changed from 1.23 to 0.85, respectively, at three months to 1.24 (*P* = 0.871) and 0.89 (*P* = 0.568), respectively, at one year.

**Conclusion:**

IOL exchange (multifocal to monofocal) to solve neuroadaptation failure in this case series resulted in significant improvements in dysphotopsia and improved distance visual function. However, UNVA worsened and patient satisfaction after exchange remained suboptimal with 77% claiming they would not repeat the lens exchange, suggesting the value of near vision spectacle independence for these patients.

## Background

The ongoing development of modern techniques for lens removal including both cataract surgery and refractive lensectomy is accompanied with higher patient expectations with intraocular lenses (IOLs) [1]. Many patients today expect good vision and spectacle independence for all distances, and this can be achieved with the implantation of multifocal intraocular lenses (MF-IOLs). However, some patients end up dissatisfied after the implantation of MF-IOLs for a variety of reasons and MF-IOL explantation may be necessary in some cases.

In the early 1990s, anterior chamber IOLs were the most frequent lenses explanted because of pseudophakic bullous keratopathy, cystoid macular edema and uveitis-glaucoma-hyphema syndrome [2]. A decade later, the main indications for the explantation changed to incorrect IOL power, decentration/dislocation and glare. Further, most explanted IOLs were posterior chamber lenses [3]. Although overall patient satisfaction with MF-IOLs is relatively high, some patients still remain dissatisfied, even when their visual acuities were excellent.

To date, there are a small number of publications that have studied the explantation of MF-IOLs in detail. Galor et al. described a series of 12 eyes that had MF-IOLs and accommodating IOLs exchanged with monofocal IOLs (MNF-IOLs), mainly because of subjective symptoms such as blurred vision, halos, glare and decreased contrast sensitivity [4]. The most common complaints in the series by Kamiya et al. were waxy vision, halos, glare, blurred vision at different distances and dysphotopsia [5]. Kim et al. found that blurred vision and photic phenomena were the most common indications for MF-IOL explantation [6]. In their series, all cases were re-implanted with MNF-IOLs, but patient satisfaction with the final outcome of the exchange was not assessed. Fernandez-Buenaga et al. reported in a national study on causes of IOL explantation in Spain with neuroadaptation failure being the main cause of explantation in patients with MF-IOLs [7].

Neuroadaptation is defined as a process in which our brain reacts to a sensory input and its capacity to adjust to any variation in this input [8]. Such a sensorial change follows the implantation of a MF-IOL to which the brain needs time to adapt to the superimposition of images and the decreased contrast sensitivity. However, when this neuroadaptation process fails, the chronicity of the symptoms is known as neuroadaptation failure [8]. Symptoms in such cases include the perception of poor quality of vision, with or without a corresponding reduction in visual acuity after excluding any refractive error or high-order aberrations. When this occurs along with dissatisfaction, one final option is to exchange the MF-IOL with another IOL. In our previous report, we studied the exchange of MF-IOLs with other MF-IOLs of a different optical design for the management of neuroadaptation failure [9]. This study investigates visual and refractive outcomes along with patient satisfaction after MF-IOL exchange with MNF-IOLs.

## Methods

This retrospective study received Institutional Ethical Board Committee approval. All patients signed an informed consent, and the study was conducted in accordance with the Declaration of Helsinki (64th WMA General Assembly, Fortaleza, Brazil, October 2013).

### Patient selection

Patients bilaterally implanted with MF-IOLs who developed neuroadaptation failure bilaterally and had an exchange with MNF-IOLs were included in the study. Patients that developed dysphotopsia or other complaints unilaterally were not considered to have neuroadaptation failure and were excluded from the study. Patients with anatomical causes for the exchange, such as lens decentration were excluded.

Selection of the explanted cases was performed in the framework of the Iberia Biobank database of explanted ophthalmic devices (UMH, Alicante, Spain). Thereafter, the clinical files of our patients were reviewed, and the data was collected in a spreadsheet (Excel, Microsoft, USA). The intraoperative information was gathered from surgical records and surgical videos. Data collected included patient age at the time of explantation, gender, primary procedure, explantation procedure, time interval between IOL implantation and explantation, implantation site, IOL design, cause for exchange, concomitant diseases, history of ocular interventions, uncorrected visual acuity for far and near, best corrected visual acuity for far and near, attempted spherical equivalent (SE), postoperative SE at three months, intraoperative and postoperative complications, and follow-up time.

The formulae used for IOL calculations for the MNF-IOL to be implanted were: SRKT and Hoffer Q using the IOLMaster (v.5.4, Carl Zeiss Meditec AG, Germany). For patients with previous refractive surgery, IOLs were calculated using the ASCRS IOL calculator (iolcalc.ascrs.org).

All primary MF-IOL implantations as well as their explantations were performed by the same surgeon (JLA) at the same institution, VISSUM Ophthalmology Institute, Miranza Group (Alicante, Spain). Explantation was decided after at least three months of neuroadaptation failure. The decision to proceed with explantation was based on significant patient complaints relating to poor quality of vision and/or quality of life (e.g., dysphotopsia, glare, halos, starbursts, etc.) caused by the implanted lens and in the absence of any residual ametropia or anatomical findings that could justify such symptoms (e.g., dry eye, posterior capsule opacification, etc.). Patients experienced a combination of different complaints, and it was hard for them to point out one neuroadaptation failure symptom causing the complaint. Patients with residual ametropia that improved more than one line of CDVA were prescribed glasses for a month’s trial. After the trial, if the patient was satisfied with his vision with glasses and all complaints disappeared, then the patient underwent a corneal refractive surgery, either LASIK or PRK depending on the case and was excluded from this study. Therefore, residual ametropia was discarded as a cause of neuroadaptation failure when patients’ dissatisfaction and symptoms remained even after correcting the ametropia either with a spectacle trial or by performing a corneal laser enhancement. Any type of irregular astigmatism was ruled out in all the patients before the first surgery which was the cataract surgery and before the second surgery which was the IOL exchange. The endothelial cell density and morphology were done before the cataract surgery in all the patients.

### Surgical technique

The aim was to preserve the capsular bag in order to re-implant into it a MNF-IOL. The optic cut technique was used to explant the MF-IOL [10]. Local peribulbar anesthesia and intravenous sedation was used in all cases; Two paracentesis of 1.0 mm and a 3.0 mm main incision were constructed. The pupil was dilated using intracameral injection of a mixture of tropicamide, phenylephrine, and lidocaine (Fydrane, Théa, France). The anterior chamber was filled with a dispersive viscoelastic (Viscoat, Alcon, USA), followed by the dissection of the IOL from the capsular bag, especially the rim of the anterior capsule using a cohesive viscoelastic (ProVisc OVD, Alcon, USA) with a 30G cannula. Using a Sinskey hook and a Lester hook (Katena, USA), the IOL was loosened from the capsular bag. Afterwards, the IOL was overlapped onto the anterior capsular rim. Subsequently, after fixing the IOL with the Sinskey hook, it was cut with IOL cutting microscissors (Katena, USA) and passed through the main incision. The cut was performed radially to the center of the IOL, followed by its extraction through the main incision using two forceps that were alternated in grasping the IOL while eliminating it from the anterior chamber. Then, the capsular bag was filled with cohesive viscoelastic (ProVisc OVD, Alcon, USA) and the MNF-IOL was implanted into the capsular bag. The procedure was finalized routinely with intracameral antibiotics (Cefuroxime 10 mg/ml, Normon, Spain). If a 10/0 nylon interrupted suture was required for incision sealing, this was then removed after three weeks of follow-up. Postoperative treatment consisted of the standard topical tobramycin combined with dexamethasone four times a day for one week and a non-steroidal anti-inflammatory three times a day for a month.

When the surgeon considered the capsular bag as unsuitable for the new IOL implantation (in relation with the integrity of the posterior capsule), a 3-piece MNF-IOL was implanted in the sulcus.

### Main outcome measures

Outcomes were evaluated at three months following the IOL exchange. The following parameters were evaluated:

- Visual and refractive outcomes

Uncorrected and best corrected visual acuities for far distance (5 m) and near distance (40 cm) were measured.

- Quality of vision evaluation

The subjective quality of vision before and after IOL exchange was evaluated using the validated Quality of Vision (QoV) questionnaire. Patients were interviewed three months after each IOL was implanted [11]. They rated 10 visual symptoms on the basis of their frequency, severity and bothersomeness. Glare, halos, starbursts, hazy vision, blurred vison, distortion, double or multiple images, fluctuation in vison, focusing difficulties, and difficulties in judging distance or depth perception were assessed. Raw data were Rasch-scaled on a 0-100 scale, with lower scores indicating better quality of vision [12].

- Subjective visual function Index-14 evaluation

The validated VF-14 questionnaire was used to evaluate the visual function three months after implantation of each IOL. It includes 14 questions about difficulties patients encounter in their activities of daily living even with glasses. The respondents chose one of five ability levels that ranged from “no difficulties” to “unable to do”. The total score was calculated by the previously described method [13]. The best score is 100, however, a score of 0 signifies the patient answered to all questions “unable to do”. In order to study the visual function in daily activities at different distances, we divided the questions into three groups. The first group contained six questions that best described far vision, the second group had three questions for intermediate vision and the third group had five questions relating to near vision. The scores for each distance were calculated using the same method. In addition, due to concerns raised over the scoring of the original VF-14, we have also performed Rasch analysis (using WINSTEPS, Version 3.93.2, Chicago, IL) to score the eight items of the refined version. This version is known as the VF-8R [14].

- Satisfaction evaluation

Patients were asked about their overall satisfaction with their near, intermediate and far vision, spectacle independence for these distances, and if the patient would repeat the surgery again either with the MF-IOL or the MNF-IOL.

### Statistical analysis

Descriptive analyses were performed using SPSS for Windows (v.18, IBM SPSS Corporation, USA). The analysis of data was based on whether data were normally or non-normally distributed. The Student’s t-test was performed to evaluate the significance of differences. The data analyzed were expressed by the mean ± standard deviation (SD) and a *P* value of less than 0.05 was considered statistically significant.

## Results

The present study included 26 eyes of 13 patients (12 females and 1 male) that underwent MF-IOL exchange. Mean patient age at the time of IOL exchange was 57.6 ± 6.7 years (range: 42–69 years). After MF-IOL explantation, the MNF-IOL was implanted into the capsular bag in 17 eyes, and in the sulcus in nine eyes. The mean time between the two surgeries was 15 ± 13 months (range: 3–45 months). The mean follow-up time after the implantation of the MNF-IOL was 33 ± 28 months. Ten eyes had posterior capsular opacification (PCO) after MF-IOL implantation and neodymium-doped yttrium aluminum garnet (Nd:YAG) laser capsulotomy had been performed. After the laser capsulotomies, patients’ satisfaction did not increase, and thus explantation was decided. Three eyes were implanted with the new MNF-IOL in the sulcus, while in the rest of the eyes, the capsular bag was considered suitable for the bag implantation with no decentration complications afterwards. One patient started to have symptoms of neuroadaptation failure before PCO developed in one eye, hence the IOL exchange was performed and then the patient underwent Nd:YAG laser capsulotomy. PCO developed in three eyes after the exchange and was treated with Nd:YAG laser capsulotomy. Residual ametropia in three eyes was treated with corneal laser enhancement before the exchange.

### Visual and refractive outcomes

The analysis of visual outcomes was preformed three months postoperatively. All patients had neuroadaptation failure associated to different photic phenomena and visual dissatisfaction despite spectacle correction of residual refractive error if there was any. The IOL models explanted and implanted are presented in Table 1. The mean uncorrected distance visual acuity (UDVA) changed from 20/38 (0.28 logMAR) with the MF-IOL to 20/36 (0.25 logMAR) with the MNF-IOL (*P* = 0.623). The corrected distance visual acuity (CDVA) improved significantly (*P* = 0.028) from 20/26 (0.12 logMAR) to 20/23 (0.06 logMAR). The spherical refractive error changed from + 0.2 D with the MF-IOL to − 0.3 D with the MNF-IOL (*P* = 0.01). The cylindrical refractive error changed from − 0.7 to − 0.6 D (*P* = 0.6), and the SE refraction changed from − 0.1 to − 0.6 D (*P* = 0.037).

**Table 1:** Patients’ data

Patient	Gender	Age (years)	Eye	MF-IOL	MNF-IOL	T (m)
1	F	53	R	RESTOR SN6AD1	SA60AT	3
			L	RESTOR SN6AD1	MN60AC	3
2	F	64	R	RESTOR SN6AD1	SN60WF	9
			L	AMO rezoom NXG1	SN60WF	9
3	F	69	R	RESTOR SN6AD1	SA60AT	3
			L	RESTOR SN6AD1	SA60AT	3
4	F	61	R	RESTOR TORIC SND1T4	MN60AC	12
			L	RESTOR TORIC SND1T3	MN60AC	12
5	F	57	R	AT LISA tri 839 MP	MN60AC	14
			L	AT LISA tri 839 MP	SA60AT	7
6	F	62	R	AT LISA tri 839 MP	MN60AC	23
			L	AT LISA tri 839 MP	MN60AC	23
7	F	54	R	Lentis LS-313 MF 30	MN60AC	7
			L	Lentis LS-313 MF 30	MN60AC	7
8	F	63	R	Lentis LS-313 MF 30	MN60AC	40
			L	Lentis LS-313 MF 30	MN60AC	40
9	M	42	R	Acri.Tec AcriLisa 366D	MN60AC	13
			L	Acri.Tec AcriLisa 366D	MN60AC	13
10	F	55	R	At Lisa tri 839 MP	SN60WF	45
			L	At Lisa tri 839 MP	SN60WF	45
11	F	52	R	RESTOR SN6AD1	MN60AC	8
			L	RESTOR SN6AD1	MN60AC	8
12	F	60	R	RESTOR SN6AD1	SN60WF	13
			L	RESTOR SN6AD1	SN60WF	13
13	F	57	R	Lentis LS-312 MF 30	SA60AT	11
			L	Lentis LS-312 MF 30	SA60AT	11

*MF-IOL* = multifocal intraocular lens that was explanted; *MNF-IOL* = monofocal intraocular lens implanted after the explantation of MF-IOL; *T (m) =* time between implantation and explantation in months; *M*  = male; *F*  = female; *R*  = right; *L*  = left

Uncorrected near visual acuity (UNVA) decreased significantly (*P* = 0.024) after the exchange from 20/47 (0.37 logMAR) to 20/62 (0.49 logMAR), while best-corrected near visual acuity (BCNVA) had no significant change (*P* = 0.130) from 20/34 (0.23 logMAR) to 20/30 (0.17 logMAR).

Most eyes (22 eyes) had a follow-up time of 1one year where the mean UDVA was 20/32 (0.23 logMAR) and mean CDVA was 20/22 (0.07 logMAR). Safety and efficacy indices changed from 1.23 to 0.85, respectively, at three months, and to 1.24 (*P* = 0.871) and 0.89 (*P* = 0.568), respectively, at one year. Of all the eyes, 15% (4 eyes) lost two or more lines of CDVA and 46% (12 eyes) gained two or more lines. The reason for the loss of CDVA in these four eyes was dry eye syndrome with punctate keratopathy, probably related to the long use of topical medication along with the prolonged follow up of these patients. Of the 26 eyes, 54% (14 eyes) were within ± 0.5 D of the SE and 81% (21 eyes) were within ± 1.0 D of the SE.

In addition, we stratified outcomes among patients that had the MNF-IOL implanted in the bag or in the sulcus. There were no statistically significant differences among the outcomes of both groups at three months. The SE did not differ remarkably in both groups at three months follow-up, being − 0.6 D for in the bag and − 0.7 D for in the sulcus (*P* = 0.935). Similarly, SE did not differ significantly between groups at the one year follow-up, being − 0.5 D for both groups (*P* = 1.000). However, at one year, the in the bag MNF-IOL group had a mean UDVA of 20/29 (0.162 logMAR) and an efficacy index of 1.06. This varied significantly to the sulcus IOL groups with a mean UDVA of 20/44 (0.34 LogMAR) (*P* = 0.021) and efficacy index of 0.63 (*P* = 0.025).

### Subjective quality of vision

QoV scores for the frequency, severity and bothersome subscales of the QoV questionnaire are displayed in Table 2. All three subscales improved significantly following the explantation of the MF-IOL and implantation of the MNF-IOL.

**Table 2:** Quality of Vision questionnaire Rasch scores of the patients before the exchange of a multifocal intraocular lens (IOL) and after the exchange to a monofocal IOL

	Preoperative score (mean ± SD)	Postoperative score (mean ± SD)	*P* value
Frequency	69.8 ± 14.4	34.5 ± 16.3	< 0.001
Severity	61.1 ± 13.6	28.5 ± 14.1	< 0.001
Bothersome	67.9 ± 14.5	28.9 ± 16.2	< 0.001

The greater the score (maximum 100), the worse the quality of vision. *SD* = standard deviation

### Visual function Index-14

The VF-14 questionnaire scores for total, far, intermediate and near are presented in Table 3. The far distance score increased remarkably after the IOL exchange, indicating a notable improvement in visual function for far distance. The visual function for near distance declined, although not significantly, as shown by the decrease in the near distance scores. Intermediate vision scores remained stable. The Rasch version of the VF-14, the VF-8R that includes only 8 questions of the original 14, showed that the mean ± SD pre-exchange score was − 1.9 ± 3.4 and this increased to − 0.2 ± 1.6 (*P* = 0.182) post-exchange. This shows worsening, although not statistically significant, in the visual function of these patients.

**Table 3:** Visual Function Index-14 questionnaire mean scores of patients before the exchange of a multifocal intraocular lens (IOL) and after the exchange to a monofocal IOL

	Preoperative score (mean ± SD)	Postoperative score (mean ± SD)	*P* value
Total score	69.1 ± 25.9	64.0 ± 11.3	0.572
Far distance	74.2 ± 24.8	90.9 ± 9.1	0.030
Intermediate distance	80.5 ± 23.9	79.5 ± 20.6	0.904
Near distance	55.4 ± 36.2	25.4 ± 30.4	0.072

The greater the score (maximum 100), the better the visual function. *SD =*  standard deviation

### Satisfaction

Patients were asked about their overall satisfaction with their vision at all distances, and the answers were analyzed (results are shown in Table 4). After the IOL exchange, we observed an increase in the percentage of patients satisfied with their far vision and a significant decrease in the percentage of satisfied patients with their near vision. When patients were asked if they would repeat the surgery with a MNF-IOL to start with, 23.1% (3 patients) answered yes. Whereas, when patients were asked if they would repeat the surgery with a MF-IOL to start with, 0% (0 patients) answered yes. Near vision spectacle independence was lost partially or totally in all cases. Results of spectacle independence and frequency of spectacle use are also shown in Table 4.

**Table 4:** The percentage of patients answering four questions regarding their overall satisfaction with their vision for different distances, their willingness to repeat the surgery, their spectacle independency and the frequency of spectacle use, all with the first multifocal intraocular lens (MF-IOL) versus the second monofocal intraocular lens (MNF-IOL)

	What was your overall satisfaction with the vision?					
	With MF-IOL			With MNF-IOL		
	Far (%)	Intermediate (%)	Near (%)	Far (%)	Intermediate (%)	Near (%)
Very good	0.0	0.0	0.0	23.1	0.0	0.0
Good	0.0	0.0	23.1	38.5	38.5	15.4
Average	53.9	61.5	38.5	38.5	53.9	23.1
Bad	30.8	23.1	23.1	0.0	7.7	7.7
Very bad	15.4	15.4	15.4	0.0	0.0	53.9

	Would you repeat the surgery?					
	With MF-IOL (%)			With MNF-IOL (%)		
Yes	0.0			23.1		
No	100.0			76.9		

	Were you spectacle independent?					
	With MF-IOL			With MNF-IOL		
	Far (%)	Intermediate (%)	Near (%)	Far (%)	Intermediate (%)	Near (%)
Yes	61.5	53.9	38.5	76.9	38.5	0.0
No	38.5	46.2	61.5	23.1	61.5	100.0

	How often you used spectacles?					
	With MF-IOL			With MNF-IOL		
	Far (%)	Intermediate (%)	Near (%)	Far (%)	Intermediate (%)	Near (%)
Never	76.9	69.2	61.5	61.5	23.1	0.0
Almost never	7.7	7.7	0.0	15.4	23.1	0.0
Sometimes	0.0	15.4	0.0	15.4	30.8	23.1
Almost always	15.4	7.7	30.8	7.7	23.1	15.4
Always	0.0	0.0	7.7	0.0	0.0	61.5

### Complications

Intraoperative complications included one eye that had a previous Nd:YAG laser capsulotomy. During the exchange, a vertical tear extended along the posterior capsulotomy with vitreous loss requiring anterior vitrectomy (the IOL was implanted within the bag eventually). Postoperative complications included mild anterior uveitis post-exchange in one eye that was medically treated successfully. In addition, a decentered IOL was observed in another eye postoperatively. The decentration was about 0.7 mm and was caused by the inadequate location of the lens inside the capsular bag that was partially retracted. Despite the dislocation, the QoV scores still improved in this patient with stable visual acuity before and after the exchange.

## Discussion

Few reports discuss the different causes behind patient dissatisfaction after the implantation of MF-IOLs. Woodward et al. [15] reported the main causes to be blurred vision and photic phenomena, and in most cases, these symptoms can be attributed to ametropia, posterior capsular opacification and dry eye syndrome. In the case series by de Vries et al. [16], 94.7% of eyes had unsatisfactory visual acuity. In the latest tenth annual survey of complications associated with foldable IOLs, Mamalis et al. reported that the second most frequent explanted IOL was the multifocal hydrophobic acrylic IOL mainly due to glare/optical aberration and incorrect IOL power [17].

In our series, we considered that neuroadaptation failure is a syndrome which includes different clinical manifestations that caused patient dissatisfaction after MF-IOL implantation. Clinically, it was difficult for many patients to single out one factor to be the main source for their dissatisfaction. Oftentimes, it is difficult for the patient to understand the concessions to be made such as decreased contrast sensitivity or some photic phenomena in exchange for having good visual acuity on all distances. This is why it is critical that the patient fully understands all probable outcomes and complications of the surgery, including the inadequate neuroadaptation process, and their possible solutions [18–20]. One solution would be IOL exchange, and in this case a monofocal IOL although this option should be considered only after considering all other treatment options. For instance, residual ametropia in some cases can be corrected by spectacles, contact lenses or laser refractive surgery. Moreover, dry eye syndrome, IOL decentration, residual ametropia and posterior capsule opacification must be ruled out before proceeding to IOL exchange.

In a previous study from our group [9], we showed that exchanging a MF-IOL with another MF-IOL of a different optical profile either in design or power is a feasible technique, while most studies [4–7, 17, 21–23] report outcomes of MF-IOLs exchange to MNF-IOLs. It has been demonstrated that complaints such as photic phenomena are more often reported by patients with MF-IOLs than with MNF-IOLs [24]. However, no statistically significant differences in halos and glare were found between refractive and diffractive MF-IOLs [25]. Our previous study [9] demonstrated that the patients treated with an exchange to another MF-IOL had better satisfaction than an exchange to a MNF-IOL.

One of the limitations of this study is that part of it was based on subjective questionnaires answered by the patients. Therefore, we suggest that the study may have a certain amount of patient expectation bias. Another limitation is the relatively low series number.

Here, we evaluated quality of vision and visual function using two validated questionnaires. The worsening, although not statistically significant, in the total score of the VF-14 questionnaire, as well as the VF-8R version, is likely due to the decline in the near visual function since we saw a significant increase in the far distance scores and therefore a significant improvement in the visual function for far distance. When patients were asked if they would repeat the surgery, almost 77% answered “no” to having the MNF-IOL to start with, and in our opinion, this is connected with the patients’ psychology and expectations. Mostly patients were dissatisfied with their near vision after the exchange since the initial expectations of near vision spectacle independence were not finally achieved. Regarding the QoV questionnaire, all three subscales, frequency, severity and bothersome, improved significantly after the exchange, meaning a reduction in the 10 symptoms included in the questionnaire.

Although the QoV questionnaire results were encouraging, visual outcomes showed a significant decrease in UNVA. Although there was a significant increase in CDVA, 15% of eyes lost two or more lines of CDVA. Such a loss in vision is unlikely to be a manifestation of the process of neuroadaptation failure as this has been shown to occur within the first six months after surgery. In addition, the accuracy of the SE refraction showed relatively poor refractive outcomes as 54% of the eyes were within ± 0.5 D and 81% were within ± 1.0 D. Despite the fact that 46% of the eyes gained two or more lines of CDVA, the abovementioned findings may suggest that the surgery is risky and should be considered carefully as a last resort to solve patients’ dissatisfaction from MF-IOLs. This once again proves the importance of proper MF-IOL selection in the first place and the competent explanation of the risk/benefits of MF-IOLs to the patients.

Another interesting observation is the sex difference. In our current study, 12 out of 13 patients were females. In addition, the majority of patients were females in our previous study (9 females out of 15 patients) regarding the exchange of MF-IOLs into other MF-IOLs of different optical profiles  [9]. Whether sex plays a role in terms of tolerance for MF-IOLs is something that we did not study, but this might be a question of interest for future studies.

In order to improve UNVA, mini monovision was targeted in many of the cases with MNF-IOLs. However, patients were still unhappy with their vision, probably due to persistent photic phenomena in some eyes or that the mini monovision was insufficient and stronger monovision should have been targeted to improve the UNVA. Furthermore, we decided to stratify the results among the patients with MNF-IOLs in the bag and MNF-IOLs in the sulcus. However, there were no statistically significant differences among the outcomes of both groups at three months. The UCVA and the efficacy at one-year follow-up showed significantly better results in patients implanted with MNF-IOL in the bag compared to those implanted in the sulcus, which shows the importance of conserving the IOL bag, if possible, in the IOL exchange procedure.

Last but not least, this subject represents a difficult issue to manage in a thoroughly satisfactory fashion. The use of validated questionnaires demonstrated that despite the exchange of the IOL that caused patients’ dissatisfaction, a majority of them remained significantly unsatisfied. This was happening in spite of the attempt to induce certain levels of monovision in a significant number of cases.

## Conclusion

This study offers a feasible solution to patients with neuroadaptation failure after MF-IOL implantation. The solution includes MF-IOL exchange with a MNF-IOL in order to improve the quality of vision and the visual function for far distance in these dissatisfied patients. However, patient satisfaction with the procedure remains low, even after MNF-IOL implantation, due to the loss of unaided near vision.

## Data Availability

Available upon request.

## Abbreviations

IOL: Intraocular lens
MF-IOL: Multifocal intraocular lens
MNF-IOL: Monofocal intraocular lens
QoV: Quality of Vision questionnaire
VF-14: Visual Function Index questionnaire
VF-8R: Rasch-revised version of the Visual Function Index questionnaire
PCO: Posterior capsular opacification
Nd:YAG: Neodymium-doped yttrium aluminum garnet
UDVA: Uncorrected distance visual acuity
CDVA: Corrected distance visual acuity
UNVA: Uncorrected near visual acuity
BCNVA: Best-corrected near visual acuity
